# Orchestration of Mitochondrial Function and Remodeling by Post-Translational Modifications Provide Insight into Mechanisms of Viral Infection

**DOI:** 10.3390/biom13050869

**Published:** 2023-05-20

**Authors:** Ji Woo Park, Matthew D. Tyl, Ileana M. Cristea

**Affiliations:** Lewis Thomas Laboratory, Department of Molecular Biology, Princeton University, Washington Road, Princeton, NJ 08544, USA; jp1381@princeton.edu (J.W.P.); mtyl@princeton.edu (M.D.T.)

**Keywords:** post-translational modifications, mitochondria, virus–host interactions, mass spectrometry, proteomics, phosphorylation, acylation

## Abstract

The regulation of mitochondria structure and function is at the core of numerous viral infections. Acting in support of the host or of virus replication, mitochondria regulation facilitates control of energy metabolism, apoptosis, and immune signaling. Accumulating studies have pointed to post-translational modification (PTM) of mitochondrial proteins as a critical component of such regulatory mechanisms. Mitochondrial PTMs have been implicated in the pathology of several diseases and emerging evidence is starting to highlight essential roles in the context of viral infections. Here, we provide an overview of the growing arsenal of PTMs decorating mitochondrial proteins and their possible contribution to the infection-induced modulation of bioenergetics, apoptosis, and immune responses. We further consider links between PTM changes and mitochondrial structure remodeling, as well as the enzymatic and non-enzymatic mechanisms underlying mitochondrial PTM regulation. Finally, we highlight some of the methods, including mass spectrometry-based analyses, available for the identification, prioritization, and mechanistic interrogation of PTMs.

## 1. Introduction

Mitochondria are dynamic organelles that control the cell’s energy metabolism, immune signaling, and lifespan [1,2]. These functions are primarily performed and regulated by proteins localized to the mitochondria. Despite the mitochondrial genome only encoding for 13 proteins, it is currently estimated that between 1100 to 1500 proteins are localized to the mitochondria [3,4]. As a result, mitochondrial health is largely dictated by expression from nuclear chromosomes and transport to the organelle; however, the functions of mitochondrial proteins are additionally fine-tuned or entirely enabled through post-translational modifications (PTMs).

PTMs provide the means to dynamically regulate protein functions by driving changes to protein abundance [5], interactions [6], subcellular or sub-organellar localization [7], and activity [8]. As methods incorporating quantitative mass spectrometry (MS) have afforded the discovery of a range of PTM types within different subcellular compartments, efforts have focused on characterizing the PTMs that are critical for cellular homeostasis or altered in various disease states [9,10,11,12,13,14,15,16,17,18,19,20]. These efforts have led to the identification of thousands of PTM-modified residues by more than 400 types of PTMs [21], deepening our understanding of the phenotypic plasticity built into the proteome through PTM-modifiable amino acids. As a crucial cellular regulatory hub, it is perhaps not surprising to find that the dysregulation of the mitochondrial proteome by PTMs is linked to many disease states, including cancer [22], aging [23], and neurodegenerative disease [23,24]. Furthermore, an accumulating body of literature points to PTM events that regulate mitochondria function and structure during infection with viral pathogens [25].

Protein post-translational modification is an important facet of virus–host interactions, allowing for the rapid toggling of protein function to either initiate host defense signaling or support the temporal and spatial coordination of a virus replication cycle. As obligate parasites, viruses must remodel the host cell proteome and metabolic output to fuel the production of their genomes, proteins, and, in certain cases, their lipid envelopes [16,19,26,27,28,29]. This is often accompanied by the modulation of host cell apoptotic signaling to prevent premature abortion of the virus replication cycle [30,31,32]. On the host side, immune signaling is propagated via phosphorylation-mediated cascades and protein shuttling on the time scale of minutes to hours [33,34]. In turn, viruses have acquired mechanisms to rapidly disable or manipulate these host defenses and ensure virus production and spread [35,36,37,38,39,40]. As a core contributor to cellular metabolism, immunity, and apoptosis, mitochondria regulation is at the cornerstone of virus replication, spread, and connected pathologies.

Here, we review PTM-based mechanisms that modulate different aspects of mitochondria function and structure. We consider different types of protein PTMs identified within the mitochondria, as well as the enzymatic and non-enzymatic mechanisms that control these modifications. Throughout the review, we highlight specific mitochondrial PTMs that have either been functionally characterized during infection or are positioned to influence viral replication. Finally, we provide an overview of available methods for the identification, prioritization, and elucidation of functional PTMs.

## 2. Mitochondrial PTMs Remodeling Mitochondrial Structure

**Figure 1:**
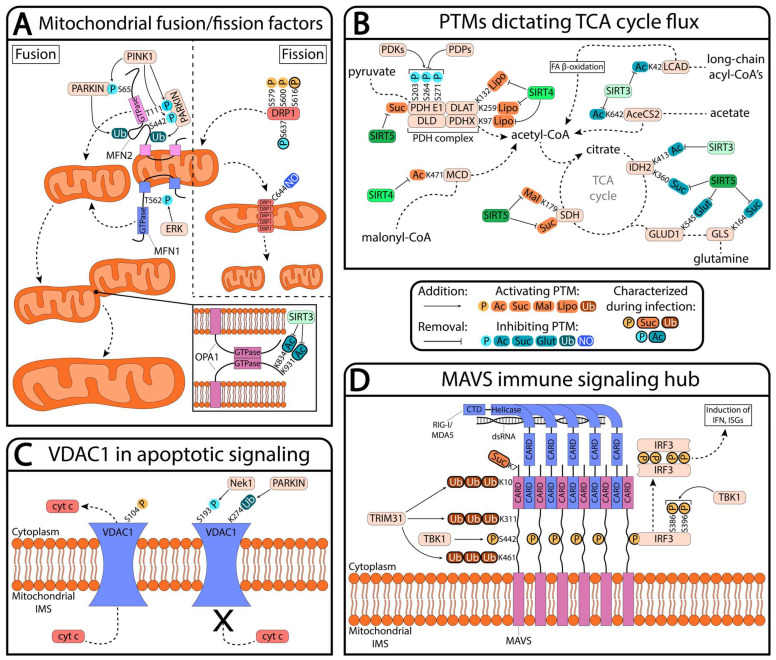
Post-translational regulation of key mitochondrial processes. (**A**) Mitochondria health is modulated by mitochondrial fusion and fission events. MFN1/2 can each promote fusion of the outer mitochondrial membrane, while OPA1 fuses the inner mitochondrial membrane. Functions of MFN1/2 and OPA1 are inhibited through a variety of mechanisms, including inhibitory acetylations on OPA1, which are removed by SIRT3 during infection (bolded PTMs have been mechanistically characterized during viral infection). Meanwhile, DRP1, a fission factor for the outer mitochondrial membrane, is phosphorylated at several residues to regulate its docking onto mitochondria. Subsequent docking promotes oligomerization and outer membrane fission, a process which is inhibited by cysteine S-nitrosylation (NO). (**B**) Mitochondria control cellular bioenergetics, and flux through their pathways are finely tuned by PTMs. Pyruvate dehydrogenase (PDH) complex activity is controlled by pyruvate dehydrogenase kinases and phosphatases (PDKs and PDPs), thus regulating the main entryway of carbon from glycolysis into the TCA cycle. Regulation of PDH and other entryways into the TCA cycle, as well as TCA cycle proteins themselves, is accomplished through a suite of activating and inhibitory lysine acylations (acetylation [Ac], succinylation [Suc], malonylation [Mal], Lipoylation [Lipo], glutarylation [Glut]). These acyl modifications are removed by mitochondrial sirtuin proteins (SIRTs), thus regulating flux through aerobic respiration. (**C**) Voltage-dependent anion-selective channel 1 (VDAC1) is an integral membrane protein on the outer mitochondrial membrane. During intrinsic apoptotic signaling, cytochrome c (cyt c) passes from the mitochondrial intermembrane space (IMS) to the cytosol across the VDAC1 channel. VDAC1 functions can be positively regulated by phosphorylation, while VDAC1 can also be inhibited through phosphorylation by Nek1 or monoubiquitination (Ub) by PARKIN. (**D**) MAVS serves as the central signaling hub for the innate immune response to RNA viruses. Activation occurs upon binding of the RNA sensors RIG-I or MDA5, facilitating IRF3 phosphorylation and subsequent dimerization. Succinylation (Suc) or addition of polyubiquitin (Ub) chains promote MAVS aggregation, making it a more effective scaffold. TBK1 phosphorylation of MAVS enables IRF3 scaffolding onto MAVS aggregates, stabilizing it for phosphorylation of IRF3 by TBK1.

Viruses frequently manipulate mitochondrial processes by remodeling mitochondrial ultrastructure and inter-organellar communication [28,41]. Mitochondrial functions depend largely on mitochondrial structure, which is regulated by both intrinsic and extrinsic factors. During homeostasis, mitochondrial dynamics are tuned to balance the rate of fission and fusion [1]. Conversely, significant bias towards either the fused or fragmented state is seen in diseases such as cancer, neurodegenerative diseases, and viral infections [42]. When mitochondria are elongated, they generally have higher mitochondrial membrane potential and metabolic output with lower apoptotic induction and mitophagy, which are all hallmarks of mitochondrial health. Alternatively, when mitochondria are fragmented, they frequently show the opposite phenotypes [43,44]. Mitochondrial fusion is mediated by the outer mitochondrial membrane (OMM) proteins mitofusin 1 and 2 (MFN1 and MFN2) and the inner mitochondrial membrane (IMM) protein optic atrophy 1 (OPA1), whereas fission is controlled by dynamin-related protein 1 (DRP1)and mitochondrial fission 1 protein (FIS1) (Figure 1A) [1]. Through regulation of the mitochondrial ultrastructure, these proteins also regulate diverse pathways independent of mitochondrial metabolism. For example, elongation of the mitochondrial network by overexpression of MFN1 or depletion of FIS1 and DRP1 has been shown to activate antiviral signaling, likely through the promotion of mitochondrial antiviral-signaling protein (MAVS) functions [45]. OPA1 additionally moonlights as a cristae structural factor, maintaining cristae width through its oligomerization [46,47]. Loss of OPA1 expression or OPA1 oligomerization has been shown to release cytochrome c, initiating apoptotic signaling [48].

Among the aforementioned mitochondrial proteins, the fusion factor OPA1 has been characterized to be regulated by PTMs (Table 1). Increased OPA1 acetylation at Lys926 and Lys931 has been observed under cardiac stress conditions and been shown to inhibit OPA1 functions, thereby suppressing fusion and contributing to mitochondrial dysfunction [49]. In the context of viral infection, OPA1 acetylation has been shown to be upregulated during human cytomegalovirus (HCMV) infection, as part of a global increase in the mitochondrial acetylome [14]. HCMV induces mitochondrial fragmentation, in part through inhibition of OPA1 function. OPA1 restricts virus production and its functions in mitochondrial fusion are inhibited through Lys834 and Lys931 acetylation. Although it has not been studied extensively, it is possible that stress-induced mitochondrial hyperfusion—dependent on L-OPA1, MFN1, and SLP-2 [50]—is mediated through OPA1 PTMs during infection with viruses that elongate mitochondria, such as Dengue virus [51]. More broadly, it remains to be determined whether the infection-induced global upregulation of the mitochondrial acetylome is connected to HCMV-linked pathologies—cardiac hypertrophy and metabolic syndrome—that also exhibit dysregulated mitochondrial acetylation [16,52,53].

Mitochondrial fission is also regulated by PTMs, such as the extensive modification of DRP1, which has been reported to be phosphorylated, ubiquitinated, SUMOylated, and nitrosylated [62]. Fragmentation of mitochondria has been shown to be driven by S-nitrosylation of DRP1 during neuronal injury [62], and phosphorylation of DRP1 by CDK1/Cyclin B has been observed to promote mitochondrial fission in mitotic cells [58]. Later studies highlighted the importance of the phosphorylation of DRP1 Ser616 and Ser637 residues for the regulation of their binding to mitochondria (Figure 1A). In agreement with this finding, the modulation of these two phosphosites has been linked with several diseases. Increased Ser616 phosphorylation and decreased Ser637 phosphorylation, concomitant with excessive mitochondrial fragmentation, have been observed in myocardial lipotoxicity [86] and several cancers [22,54,55]. In the context of viral infection, both hepatitis B and C viruses have been shown to induce mitochondrial fragmentation by elevating DRP1 Ser616 phosphorylation [56,57]. In both viral infections, DRP1-mediated fission led to mitophagy, thereby attenuating virus-induced apoptosis. In HCV infection, it has been further shown that DRP1 knockdown decreased virus secretion, glycolysis, and cellular ATP level, while enhancing innate immune responses. Although DRP1 phosphorylation studies have mainly focused on Ser616 and Ser637 phosphorylations, it has been found that, during HIV-1 infection, DRP1 Ser620 and Ser629 residues are also phosphorylated [87]. These sites remain to be functionally characterized. Considering that overexpression of DRP1 inhibits the loss of mitochondrial membrane potential and apoptotic cell death by HIV-1 viral protein R (Vpr) [88], it is possible that DRP1 activity is targeted by HIV-1 via the phosphorylation of these uncharacterized sites. Of note, this study also found that Vpr increases the bulging of mitochondria-associated membranes (MAM), which are areas of contact between the ER membrane and the OMM [88].

Mitochondria, like all other organelles, do not function in isolation, but instead form dynamic inter-organellar networks through membrane contact sites (MCSs). Distance between the organelles in MCSs can range from 10 nm to 100 nm [89], and these contacts are composed of several different classes of proteins with distinct roles [90]. Structural proteins act as tethers and spacers in the MCS to stably hold the organelles together at a certain distance. This is most often achieved through homotypic or heterotypic interactions between two proteins on the opposing membranes [90]. This enables the shuttling of metabolites and signaling molecules between organelles without fusion of the organellar membranes. For example, ER–mitochondria contacts facilitate lipid transport [91], Ca^2+^ transport [92], ROS signaling [93], and autophagy [94].

MCSs are altered in several diseases such as cancer, Alzheimer’s disease (AD), and diabetes, as well as viral infections [95]. HCMV remodels all major organelles during its replication cycle [96], and a recent study has highlighted that HCMV infection globally increases MCS protein abundances [97]. This stands in contrast with the fine-tuning of specific MCSs during infection with HSV-1, as well as the overall decrease in MCSs for influenza A and HCoV-OC43. These findings are in line with the understanding that each virus uniquely alters organelle structure–function relationships to aid its replication. For example, HSV-1 has been previously reported to increase peroxisome biogenesis [98], and HCoV-OC43 has been shown to restructure the ER to form the double-membraned vesicles used for viral replication and capsid assembly [99]. HCMV has been found to induce a previously unreported MCS structure termed mitochondria–ER encapsulation (MENC), which is supported by tethering proteins VAP-B and the relocalized PTPIP51 [97]. The molecular regulators of MENC formation are not yet known, but PTM-based regulation may aid in the formation of this structure, as has been observed for MCSs in the past. For example, PTPIP51 phosphorylation events have been reported to impact several protein–protein interactions [100,101,102], and contact between mitochondria and vacuole—known as a vacuole and mitochondria patch (vCLAMP)—has been shown to be regulated by phosphorylation of Vps39 in yeast [5]. Additionally, phospho-FFAT domains have been shown to regulate ER–endosome contact depending on phosphorylation state [103]. With the accumulating knowledge that viral infections induce numerous protein translocation events [34], it is likely that PTMs are more broadly involved in inter-organellar communication, as well as intra-organellar remodeling.

## 3. Mitochondrial PTMs Regulating Cellular Processes

### 3.1. Metabolism

Given that a primary role of mitochondria in cell survival is to regulate metabolic pathways to support bioenergetics, a main functional interaction between viruses and mitochondria is at the interface of metabolic control. Viruses rely on host metabolites for energy and precursors for their genomes, proteins, and lipid envelopes. Hence, the temporal rewiring of metabolic pathways is a feature of many viral infections. An infection-induced alteration is the upregulation of glycolysis, a major carbon entry point into the TCA cycle and oxidative phosphorylation pathway. Upregulated glycolysis has been observed during infection with diverse viruses, including Kaposi’s sarcoma herpesvirus (KSHV) [104], human cytomegalovirus (HCMV) [26,27], influenza A [105], HIV-1 [106], Dengue [107], and SARS-CoV-2 [108]. Some of these viruses induce a “Warburg-like effect” [109], similar to the metabolic phenotype characteristic of cancers, with this aerobic glycolysis enabling rapid energy production and shunting of carbon into production of biomass for cellular proliferation. The resulting pyruvate is not further oxidized by mitochondrial pathways but is instead reduced to lactate in the cytosol, thereby regenerating NAD^+^. Increased lactate production is known to dampen immune signaling [110], and to also contribute to an anti-inflammatory tumor microenvironment through the modulation of infiltrating immune cells [111,112,113,114,115]. It is likely that this immunosuppressive outcome is also beneficial for preventing interference with viral replication. Furthermore, the metabolic reprogramming caused by certain infections is thought to be linked to the oncogenic or oncomodulatory properties of these viruses, such as for KSHV [104] and HCMV [116].

A number of metabolomics studies have demonstrated that virus infections can promote flux through the TCA cycle, as shown for HCMV [27], HSV-1 [117], and SARS-CoV-2 [118]. HCMV increases TCA cycle intermediates, both for efflux of citrate into fatty acid synthesis pathways for production of its lipid envelope [27], as well as increased energy production through respiration [119]. HCMV additionally encodes a viral protein, pUL13, which promotes electron transport chain function through cristae remodeling [28]. However, this upregulation of oxidative phosphorylation seems to make HCMV the exception, rather than the rule. HSV-1 increases anaplerotic influx into the TCA cycle for nucleotide biosynthesis to support genome replication [117], not bioenergetics. During infection with SARS-CoV-2, glucose influx to the TCA cycle is upregulated, but carbon oxidation through the TCA cycle is repressed [118]. Given that lipid synthesis is required for completion of the SARS-CoV-2 replication [120], it is possible that carbon from the TCA cycle is directed into lipid anabolic pathways via citrate, much like for HCMV. Some giant DNA viruses even encode TCA cycle enzymes in their genomes [121], presumably to spur metabolic flux through these pathways. Despite many virus infections leading to reduction of oxidative phosphorylation as a means of energy production, the TCA cycle still serves as an important hub for carbon efflux into various anabolic pathways. Perhaps an additional reason why many viral infections lead to reduced electron transport chain activity is to temper reactive oxygen species (ROS) production, which has been shown to activate innate immune signaling through NLRP3 inflammasomes [122]. Through the above-mentioned upregulated glycolysis and carbon flux towards biosynthetic pathways at the expense of electron transport chain activity, the virus requirements for energy production and biomolecule generation can be met without the deleterious impacts of aerobic respiration.

Protein PTMs are known to be critical switches of these metabolic pathways [123]. Phosphorylation-mediated regulation of the pyruvate dehydrogenase complex (PDH) activity by kinases and phosphatases (PDKs and PDPs) adjusts pyruvate flux into oxidative pathways, thus serving as a gatekeeper for mitochondrial metabolism (Figure 1B) [29]. PDH phosphorylation has been detected in different viral infections: HIV-1 [87], HSV-1 [124], influenza A [125], and DENV [126]. Phosphorylation of PDHA1, the E1a subunit of PDH, at Ser203, Ser264, and Ser271 leads to the inactivation of the complex [75,76,77,78]. These three sites are all located in the highly conserved phosphorylation loops A and B of PDH, which fold into ordered conformation upon thiamin diphosphate (ThDP) binding [127,128,129]. Even in the presence of ThDP, phosphorylation at these sites induces a disordered structure of the loops due to the steric clashes caused by the phosphate groups, thereby inactivating PDH [76,127]. Interestingly, all three known PDHA1 phosphosites were found during HSV-1 infection [124], and Ser203 phosphorylation has been found in both HSV-1 and influenza A infections [124,125]. Additionally, Ser241 phosphorylation, a previously uncharacterized phosphosite, was found in HIV-1 infection [87]. Regulation of these phosphosites may constitute a viral mechanism for either disabling PDH to promote aerobic glycolysis or activating PDH to facilitate entry and subsequent efflux from the TCA cycle into biomolecule precursors [117]. Consistent with this, PDH activity has been observed to markedly decrease in mice infected with influenza through a yet unknown mechanism [130]. Other PTMs have also been shown to modulate PDH activity, including succinylation and lipoylation [79,80], and it remains to be seen whether these PTMs are altered during viral infections.

Components of the TCA cycle are also extensively regulated by PTMs, as seen during infection with HCMV [16]. Malate dehydrogenase (MDH) has been shown to be activated through acetylation in high glucose environments [131], while acetylation of succinate dehydrogenase (SDH) decreases its enzymatic activity by preventing substrate entry into the active site [132,133,134]. Further, phosphorylation of NADH dehydrogenase (ubiquinone) flavoprotein 2 (NDUFV2) has been demonstrated to be essential for sufficient ATP production for cell survival [135]. In addition to the TCA cycle and oxidative phosphorylation, mitochondrial proteins involved in fatty acid metabolism are also regulated by PTMs. Dysregulated lipid metabolism is a hallmark of many cancers and viral infections, as fatty acids are used for membrane biogenesis, intracellular signaling, and production of acetyl-CoA to fuel the TCA cycle [27,120,136,137]. Acetyl-CoA carboxylase (ACC) is an enzyme involved in de novo fatty acid synthesis that irreversibly carboxylates acetyl-CoA to malonyl-CoA [138]. It has been reported that, in nutrient abundant conditions, ACC2 is hydroxylated at proline 450 by prolyl hydroxylase 3 (PHD3) to increase its activity, thus repressing fatty acid oxidation [139]. The global changes in mitochondrial protein acetylation that are starting to be observed during viral infections may broadly direct metabolic flux away from oxidative phosphorylation and towards anabolic pathways, such as de novo lipid synthesis [16,27].

### 3.2. Apoptosis

Most viruses have acquired mechanisms to inhibit apoptotic signaling to allow time for viral replication, while occasionally inducing apoptosis late in replication to facilitate dissemination of virions [30,31,32]. Mitochondria function as central mediators of both intrinsic and extrinsic apoptotic signaling pathways [140,141,142]. In the intrinsic pathway, apoptotic signals activate pro-apoptotic B cell lymphoma 2 (BCL-2) proteins, which subsequently activate the pro-apoptotic effectors BAX and BAK, leading to mitochondrial outer membrane permeabilization (MOMP) [143]. MOMP irreversibly commits the cell to death in a caspase-independent manner by causing efflux of several small pro-apoptotic molecules such as cytochrome c [144,145,146]. In the extrinsic pathway, death receptor ligands activate cell surface death receptors such as TNF-related family receptors, resulting in activation of caspase-8 [140,141]. Active caspase-8 advances apoptosis by either inducing a cascade of executioner caspases or translocating the pro-apoptotic protein tBid to the mitochondria, thereby intersecting with the intrinsic pathway and promoting MOMP [140,147,148]. 

Both intrinsic and extrinsic apoptotic signaling pathways are regulated by PTMs. Outside of the mitochondria, caspases can be phosphorylated by various kinases to both suppress and activate functions in apoptosis [149,150,151,152,153]. For example, the pro-survival kinase Akt has been shown to phosphorylate human caspase-9 at Ser196 to inhibit its activity, resulting in the failure of apoptotic induction even upon cytochrome c release from the mitochondria [152]. Caspase-3 activity, on the other hand, has been shown to be enhanced by phosphorylation by PRKCD. This phosphorylation increases the proteolytic activity of caspase-3, thereby amplifying the transmission of apoptotic signals [151]. Another important pro-apoptotic protein, BAX, is regulated by the ubiquitin/proteasome pathway. BAX undergoes ubiquitin-dependent degradation mediated by E3 ligases PARKIN and IBRDC2, thus preventing initiation of MOMP to induce apoptosis [154].

Most PTMs shown to affect apoptotic signaling operate through regulating the multimerization and relocalization of cytoplasmic proteins; however, mitochondrial protein PTMs further regulate initiation of intrinsic apoptotic signaling. Cytochrome c has been found to be acetylated on Lys53 in human prostate xenograft samples, inhibiting its function in apoptotic induction [155]. Another study has shown that DRP1 is SUMOylated during apoptosis in a BAX/BAK-dependent manner [156,157,158]. The mitochondrial-anchored RING-finger-containing protein (MAPL) SUMOylates DRP1 at ER–mitochondria contact sites to facilitate mitochondrial fission and calcium transfer, thus ultimately leading to cytochrome c release and apoptosis [158].

Interestingly, some apoptosis-related mitochondrial PTMs characterized in other pathologies are also present in different viral infections [159,160,161]. A notable example is voltage-dependent anion channel (VDAC) (Figure 1C). VDAC is an OMM protein that regulates the transfer of essential molecules such as calcium, NADH, ADP, ATP, and other metabolites [162,163,164,165,166,167]. It exists in three isoforms (VDAC1, 2, and 3) in mammals [168], and phosphorylation of their serine, threonine, and tyrosine residues have been observed by mass spectrometry in all three isoforms [169,170]. VDAC dysfunction has been reported in various neurodegenerative diseases such as Alzheimer’s disease [171,172], Down’s syndrome [172], and familial amyotrophic lateral sclerosis (ALS) [173,174]. Recent studies have shown that specific PTMs, such as VDAC1 and VDAC3 deamidations, are enhanced and can regulate channel behavior in ALS cell lines [175,176]. Moreover, VDAC1 phosphorylation is dynamically regulated in a variety of human cancers, suggesting a role in tumorigenesis [177]. In the context of viral infections, hepatitis B virus (HBV)-encoded protein HBx has been shown to bind VDAC3 and alter the mitochondrial membrane potential, as well as activate STAT-3 and NF-κB [178,179]. Given recent findings that show over-oxidation of VDAC3 methionine and cysteine residues in both rat and human liver [180,181], it is possible that liver-associated pathologies in various disease states, including viral infections, are driven in part by VDAC3 PTMs [182,183]. Phosphorylation of VDACs has additionally been observed by mass spectrometry during infection with herpes simplex virus 1 (HSV-1), human immunodeficiency virus 1 (HIV-1), and influenza A [87,124,125]. Although not functionally characterized during viral infection, VDAC1 Ser104 phosphorylation was observed in all three studies, a phosphorylated residue previously shown to stabilize VDAC1 and induce apoptosis [82,83].

VDAC1 and VDAC3 have been shown to restrict replication of HSV-1 by facilitating cytosolic release of mitochondrial DNA (mtDNA), thereby increasing the cellular immune response [184]. This indicates that MOMP-induction by VDACs is functionally relevant to HSV-1 replication. Since VDAC oligomerization is crucial for mtDNA release, the observed VDAC phosphorylations may suppress its oligomerization, leading to inhibition of apoptosis. In contrast to Ser104 phosphorylation stabilizing VDAC1 functions, phosphorylation at Ser193 by Nek1 closes VDAC1, thus preventing cytochrome c release into the cytosol (Figure 1C) [84]. Additionally, monoubiquitination at Lys274 by PARKIN suppresses apoptosis [185]. The observed VDAC phosphorylation events in viral infections may also regulate channel conformation and VDAC1 stability during infection, constituting an interface of host–pathogen competition over control of intrinsic cell death pathways.

### 3.3. Immune Signaling

Mitochondria orchestrate both adaptive and innate immune signaling through various mechanisms. As the driver of cellular bioenergetics, mitochondria most prominently influence the immune system through modulation of immune cell metabolism [85,186]. For example, the hexosamine biosynthetic pathway (HBP) is a glucose metabolism pathway that produces uridine diphosphate-*N*-acetyl glucosamine (UDP-GlcNAc), a precursor of *O*-linked and *N*-linked glycosylations [187]. UDP-GlcNAc is then utilized to synthesize glycans by adding *O*-linked *N*-acetyl glucosamine (*O*-GlcNAc) to mitochondrial proteins [188,189,190,191]. This specialized PTM is called *O*-GlcNAcylation and it has been shown to be involved in various roles, such as maintaining immune cell functions [192,193,194]. Additionally, glutaminolysis is another critical metabolic pathway for immune cell development and response. Glutamine is required for the induction of IL-1 by macrophages upon lipopolysaccharide (LPS) stimulation [195]. Furthermore, glutaminase inhibition has been shown to decrease the production of nitric oxide by Bacille Calmette-Guérin (BCG)-activated macrophages [196,197].

Aside from the regulation of immune cell metabolism, mitochondria act as central hubs of innate immune signaling across cell types. MAVS is located on the OMM and is activated by the cytoplasmic pattern recognition receptors (PRRs) RIG-I and MDA5 [198,199,200,201]. Upon detection of viral dsRNA, N-terminal tandem caspase activation recruitment domain (2CARD) of RIG-I or MDA5 undergoes homo-oligomerization, which in turn induces MAVS CARD filament formation [202,203,204]. This filament formation is necessary for the recruitment of TRAF molecules and for IRF3 dimerization to activate the downstream signaling pathway [204]. MAVS-mediated innate immune signaling is not restricted to the cytoplasmic viral RNA sensing pathway, as numerous studies have revealed that RNA and DNA sensing pathways display complex crosstalk [205,206,207,208,209,210]. It has been shown that RNA polymerase III can use cytosolic DNA to synthesize dsRNA, which serves as a ligand for RIG-I like receptors (RLRs) to induce IFN-B production through the MAVS signaling pathway [207]. Moreover, stimulator of interferon genes (STING), the ER-resident protein that serves as an immune signaling hub in the cGAS–STING DNA sensing pathway, has been suggested to interact with RIG-I and MAVS in a super-complex that is stabilized during viral infection [205,206,208]. Mitochondrial dynamics and ER–mitochondria tethering have been shown to directly impact the MAVS–STING interaction, as well as STING activation [97], consequently affecting downstream immune signaling activity [45]. 

MAVS expression and ability to recruit signaling factors have been shown to be altered by various PTMs (Figure 1D) [45,211,212,213]. Most prominently, MAVS is phosphorylated at Ser442 by the kinase TBK1, a moiety which is required for the binding of the immune signaling factor IRF3 to MAVS. Upon IRF3 scaffolding onto P-Ser442 of MAVS aggregates, TBK1 phosphorylates IRF3, enabling IRF3 dimerization and subsequent nuclear translocation for induction of interferon (IFN) and interferon-stimulated genes (ISGs) [33]. As IRF3 relies on both MAVS phosphorylation and its aggregation for activation, PTMs which influence MAVS aggregation will also regulate its function as a signaling hub. During infection with Sendai virus and vesicular stomatitis virus, TRIM31-based poyubiquitination of MAVS at Lys10, Lys311, and Lys461 has been shown to promote its aggregation and downstream immune signaling [68]. Succinylation of MAVS at Lys7 has a similar effect in promoting MAVS aggregation [69]. Phosphorylation by Nemo-like kinase (NLK), on the other hand, has been shown to suppress antiviral signaling by promoting degradation of MAVS [70].

Additional uncharacterized MAVS phosphorylation events have been reported in HSV-1, HIV-1, and influenza A infections [87,124,125,159]. One such phosphosite, MAVS Ser222, has been reported after stimulation of human myeloid leukemia cells with TNF, which was an unexpected finding since MAVS is not known to be a part of TNF signaling pathways [214]. Next, MAVS Ser152 phosphorylation has been reported in a study that investigated the impact of iron deficiency in neuronal cells [215]. Acute iron deficiency in HT-22, a hippocampal-derived neuronal cell line, resulted in the increased phosphorylation of proteins involved in inflammatory pathways, including MAVS. The consistent observation of these sites across pro-inflammatory conditions suggests that they could serve roles in modulating MAVS function. It will be interesting to see if MAVS is additionally regulated by inhibitory PTMs, perhaps added directly by viral proteins, or if virus antagonism of MAVS is primarily through obstruction of the aforementioned gain-of-function PTMs on MAVS.

## 4. Enzymatic and Nonenzymatic Regulation of Mitochondrial PTMs

Post-translational regulation of protein function is often dictated by the competition of PTM “writers” and “erasers” over the protein substrate: phosphorylation through kinases and phosphatases, acetylation through acetyltransferases and deacetylases, and ubiquitination through ubiquitin ligases and deubiquitinases [216]. This paradigm extends to the mitochondrial proteome for many PTMs, such as the pyruvate dehydrogenase kinases and phosphatases regulating PDH activity [77,217,218,219]. However, ROS induces PTMs on proteins via nonenzymatic mechanisms [220]. Many viruses alter redox state during their replication cycles (reviewed in [221]), but the extent to which this influences ROS-induced PTMs on host and viral proteins is not well understood. Further, the addition of mitochondrial acetylation and other acylations is believed to occur primarily through nonenzymatic mechanisms (reviewed in [222,223]). In vitro experiments have demonstrated that the slightly alkaline pH of the mitochondrial matrix improves the nucleophilicity of lysines, enabling it to spontaneously react with the highly abundant acetyl-CoA pool [224]. In cells, this may be further promoted by a semi-enzymatic mechanism, whereby lysines acquire acetylation via donation from a transiently acetylated proximal cysteine [225]. Through these mechanisms, non-enzymatic acetylation regulates mitochondrial protein stability and enzymatic activities [131].

In contrast with the diverse regulatory roles for protein acetylation on non-histone proteins in the nucleus, mitochondrial protein acetylation has been found to be inhibitory in nearly every mechanistic study, thus serving as a “nonenzymatic lesion” on proteins [226]. Some have called into question the functional capacity of these modifications, as absolute quantification studies have reported a sub-1% stoichiometry for many acetyllysine modifications on their target protein [226,227]. While many such modifications are likely negligible to protein function, it is possible for regulation at that site to have functional importance without a high modification stoichiometry (>10%), especially if that protein is part of multiple protein complexes and only one complex is being inhibited. Many of these acetyllysine sites can also be modified by other acyl modifications [228], likely leading to an underestimation of the total acylation at each lysine. Furthermore, the concurrent low-stoichiometry modification of many lysines on each individual protein, as well as on several proteins within the same metabolic pathway, may cumulatively inhibit flux through that pathway.

Removal of acetylation and other acyl modifications is in fact important for regulating mitochondrial metabolism. This is achieved through a more canonical, enzymatic route: mitochondrial sirtuin (SIRT) proteins serving as “erasers”. Sirtuins are conserved across domains of life, maintaining cellular homeostasis through regulation of a diverse array of lysine acylations [229,230,231]. Mammalian sirtuins regulate pathways throughout the cell, with three of the seven mammalian sirtuins localizing to the mitochondria: SIRT3, SIRT4, and SIRT5 [232,233]. SIRT3 functions as the primary mitochondrial deacetylase [226,234], while SIRT5 preferentially removes acidic acyl modifications, such as lysine malonylation [235], succinylation [236], and glutarylation [237]. SIRT4 is able to remove the large, branched modification lipoylation [80,234], while also displaying activities against various glutarylations [238], biotinylation [80], and acetylation [71]. In this way, sirtuins specialize in the protein substrates that they regulate as well as the acyl moieties on those substrates.

Mitochondrial sirtuins broadly regulate mitochondrial homeostasis, primarily by directing flux through metabolic pathways (Figure 1B). Acetylation decorates metabolic proteins and inhibits flux through these pathways, a stress which is relieved by SIRT3 [239,240,241,242]. To protect against the consequences of hyperactive oxidative phosphorylation, SIRT3 activates ROS-mitigating systems by deacetylating the mitochondrial superoxide dismutase MnSOD [243,244]. In contrast with the promotion of aerobic respiration by SIRT3, SIRT4 and SIRT5 restrict aerobic respiration. SIRT4 inhibits carbon entry into the TCA cycle by removing lipoylation from pyruvate dehydrogenase [80], further decreasing acetyl-CoA flux through oxidative pathways through the regulation of malonyl-CoA decarboxylase [71,245]. By promoting the catabolism of leucine [238], SIRT4 may additionally impact mTOR-mediated cell growth by removal of one of its key agonists [246]. SIRT5 restricts carbon entry in the TCA cycle by removing activating succinylation marks [79], favoring fatty acid β-oxidation for energy production [247]. SIRT5 further slows TCA cycling by removing succinylation and malonylation from succinate dehydrogenase [79,81], while paradoxically activating the TCA enzyme isocitrate dehydrogenase [66]. The disparate effects of acidic and branched acyl modifications on metabolic protein function stand in contrast to the nearly pan-inhibitory impact of acetylation on these same proteins.

As guardians of mitochondrial bioenergetics, mitochondrial sirtuins are implicated as drug targets across disease states characterized by metabolic dysregulation. Viruses cause ~15% of cancers worldwide [248], with their promotion of a Warburg-like metabolic phenotype believed to be partially responsible [116]. Depending on the specific metabolic needs of the cancer, mitochondrial sirtuins have a dichotomous function in either promoting tumorigenesis or acting as tumor-suppressors. SIRT3 functions as a tumor-suppressor in characteristic aerobic glycolysis-dependent tumors by maintaining flux through aerobic respiration, a known characteristic of non-proliferating cells [249,250,251]; however, SIRT3 is also overexpressed in certain tumors [252]. Through its inhibition of aerobic respiration [81], as well as its protection of glutamine anaplerosis in mitochondrially active tumors [64,253], SIRT5 is frequently found to be pro-tumorigenic; however, its desuccinylase activity restricts tumors characterized by mitochondrial hypersuccinylation [254]. Meanwhile, through decreasing acetyl-CoA and glutamine influx into the TCA cycle, SIRT4 is positioned to restrict the same mitochondrially active tumors that benefit from the functions of SIRT3 and SIRT5 [255,256].

Despite many metabolic similarities to cancers and findings showing that mitochondrial sirtuins impact replication of various viruses [26,27,104,105,106,107,108,117,118,120,257,258], sirtuins have been comparatively understudied during infection. SIRT5 has been shown to promote Sendai virus and SARS-CoV-2 infections [69,259], possibly through an indirect effect of SIRT5 on cellular metabolism [259]. Then, the avian Newcastle disease virus induces mitophagy and degrades the antiviral SIRT3 protein enroute to a Warburg-like metabolic phenotype [260]. SIRT3 additionally exerts an antiviral effect against HCMV through regulation of the mitochondrial ultrastructure [14]. SIRT3 deacetylates the fusion factor OPA1 to derepress its function (Figure 1A), interfering with the fragmentation of mitochondria that is characteristic of HCMV infection. Albeit through a regulation of mitochondrial morphology and not through the direct regulation of metabolic proteins, the functional consequence of OPA1 regulation is an alteration of mitochondrial metabolism. Consistent with the role of mitochondrial sirtuins in cancers, their role in either promoting or restricting viral replication will likely result from the specific metabolic program induced by the virus.

## 5. Methods for Identifying and Determining the Mechanism of PTMs

**Figure 2:**
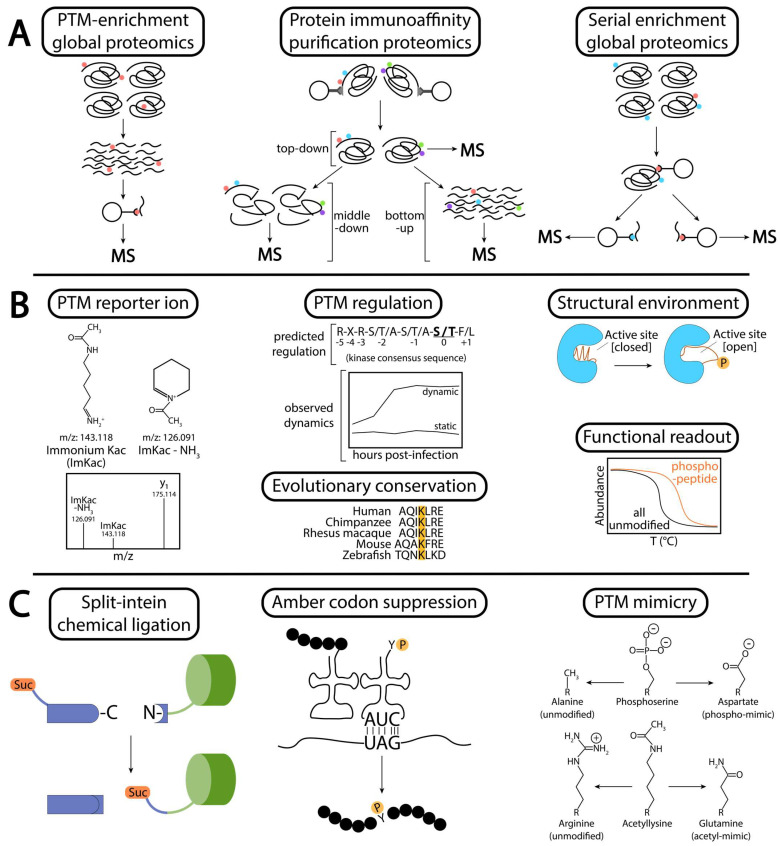
Techniques to identify and functionally characterize PTMs. (**A**) Methods for identifying the PTM-modified proteome and PTM crosstalk. PTM-enrichment global proteomics through a “bottom-up” approach of full enzymatic digestion of cellular protein enables optimal proteome coverage of a particular PTM, while protein immunoaffinity purification followed by proteomics allows identification of multiple types of PTMs on a target protein. Semi-digested protein (“middle-down”) or undigested protein (“top-down”) can be analyzed by mass spectrometry to identify patterns of PTM co-occurrence or competition, suggesting PTM crosstalk. PTM correlations can be globally profiled by serial enrichment of PTMs. (**B**) Methods for predicting highly functional PTMs. Use of reporter ions for the modified amino acid can bolster confidence in a PTM assignment. Use of prediction tools for known “writers” and “erasers” of a PTM can suggest regulation at that site, while directly observing change in a PTM abundance across biological conditions makes regulation of the site evident. PTM-modifiable residues that are highly conserved suggest that PTM regulation at that site experiences a positive selective pressure. Further, integration of PTM sites with known or predicted structures can suggest changes in protein conformation upon PTM addition, such as the phosphorylation of a flexible region evicting it from the protein’s active site and enabling its enzymatic activity (phospho-activation loop). Finally, incorporating a functional readout during data acquisition can immediately predict changes in protein function from a PTM. Thermal proteome profiling paired with PTM-omics can identify PTM-modified proteoforms with distinct thermal denaturation curves, suggesting a change in that protein’s interactions or biochemical stability upon addition of the PTM. (**C**) Techniques for mechanistically interrogating PTMs. Use of chemical ligation technologies, such as a split-intein, allows for direct addition of PTM-modified peptides onto the termini of proteins. Through a semi-synthetic biology approach, cells expressing a foreign tRNA recognizing amber codons (the least common stop codon in humans) and a corresponding tRNA ligase can be used to directly incorporate PTM-modified amino acids at an amber codon within a gene’s coding sequence. Finally, due to ease of use and applicability to higher organisms, many researchers opt for site-directed mutagenesis of the target protein to amino acids which mimic the PTM-modified form or the constitutively unmodified form.

A range of methods can be deployed for identifying PTMs, most commonly through antibody-based detection or by mass spectrometry. Mass spectrometry-based (MS) proteomic approaches enable high-throughput identification and quantification of protein PTMs, in conjunction with the determination of the specific residues that are modified [261,262]. Bioinformatic tools even allow for the identification of novel types of PTMs without any a priori information [263]. Given that only a fraction of each cellular protein will exist in a modified form, enrichment is frequently required for extensive coverage of the PTM-modified proteome. Affinity purification using antibodies or cation beads can be used to enrich PTMs of interest [264,265,266,267,268], typically after protease digestion of cellular proteins into peptides, using a strategy known as “bottom-up” proteomics (Figure 2A). Use of titanium dioxide cation beads has been deployed for global profiling of the phosphoproteome during different types of viral infection [15,19,87,125], while antibody-based methods have monitored other types of PTMs, including acetylation [16], succinylation [20], and ubiquitination [17,269]. These PTM-specific enrichment methods have proven optimal for broad coverage of certain PTMs of interest within the cellular proteome. Additionally, immunoaffinity purification of a specific protein, followed by MS analysis, can allow for the simultaneous identification of multiple types of PTMs on the isolated protein [270]. Analysis of the intact or partially digested protein—“top-down” or “middle-down” proteomics—afford the identification of co-occurring PTMs that may participate in “crosstalk” mechanisms [271,272,273,274], whereby one modification either positively or negatively regulates another modification. For example, PINK1-induced phosphorylation scaffolds Parkin binding and the subsequent ubiquitination of target proteins (Figure 1A) [73,74]. Swaney et al., (2013) took a creative approach when they performed enrichment for ubiquitination at the protein level followed by the subsequent digestion and enrichment of phosphopeptides, thus profiling, on a large-scale, the co-regulation of ubiquitination and phosphorylation [275]. Other similar serial enrichment approaches have been deployed for the deep analysis of multiple PTMs from a single sample [276]. Determining the influence of one modification on other modifications on the same or different proteins has proved to be technically challenging (reviewed in [277]), but it is likely to be a common principle of protein regulation.

While these MS-based approaches have substantially enriched the knowledge of PTM types and sites [13,14,15,17,18,19,20,87,125], the functional relevance of most of these modification sites has remained unknown. Considering the low-throughput nature of the mechanistic characterization of PTMs, several groups have endeavored to prioritize the most-likely functional PTMs. For example, Ochoa et al., (2020) mined the publicly available phosphoproteome datasets, totaling a staggering 221,236 human phosphosites [278], and prioritized sites based on four criteria (Figure 2B): (1) quality of MS evidence for the PTM, including identification of diagnostic reporter ions for the PTM in question [279,280]; (2) PTM regulation, inferred by comparison with local sequence motifs for the PTM writer [281,282] or directly found to be dynamic by comparison between biological conditions [283], (e.g., infected versus uninfected cells); (3) structural environment, determined either experimentally or computationally predicted (e.g., AlphaFold [284]), which can point to PTMs that are solvent accessible or that may modify protein structure enroute to functional regulation [285]; and (4) evolutionary conservation, as this suggests a selective pressure for maintenance of PTM-based regulation at that site [286,287]. Further, global analysis of the PTM can be paired with a functional readout during data acquisition. For example, the thermal stability of proteins upon heat denaturation can be used to predict changes in protein complex association [288,289,290], that, when integrated with phosphoproteome analysis, can directly predict a functional outcome of specific phosphorylated sites (Figure 2B) [291,292]. These types of analyses can be integrated to increase confidence in the regulatory potential for the identified PTMs.

Another consideration is that the PTM-modified proteoform must be mechanistically interrogated by comparison with the unmodified proteoform. There are different approaches available, each with relative strengths and weaknesses (Figure 2C). Early approaches involved chemical ligation of a PTM-modified peptide into a target protein for in vitro studies of histones [293,294]. Intein-based protein splicing has allowed for incorporation of PTM-modified peptides into native proteins in cell culture, proving invaluable for dissecting the histone code [295]. Like most ligation technologies, this approach is limited to the introduction of the modifications at the termini of the proteins of interest. To incorporate modifications throughout the primary sequence of a protein, commonly utilized techniques are genetic code expansion and site-directed mutagenesis. Genetic code expansion through amber codon suppression allows one to incorporate PTM-modified amino acids into the native context in cells and in vivo [296,297,298], but suppression of endogenous amber codons leads to nonspecific incorporation throughout the proteome [299,300]. Mutagenesis of the residue identified to carry the PTM to amino acids that either mimic the modified version or block the modification is a widely used approach for assessing the molecular functions of specific sites. While site-directed mutagenesis approaches have proven valuable for the discovery of many of the PTM functions currently known, caution must be exerted when interpreting the results, as the mimics do not perfectly recapitulate the structure of the modified residue. Additionally, these approaches give rise to a protein that is ~100% modified, which is typically much higher than the stoichiometry of the modification in vivo. While the assays may demonstrate that a PTM is sufficient to induce a particular phenotype, the observed phenotype may be enhanced compared to the effect generated from the native PTM.

## 6. Concluding Remarks

Mitochondria dysregulation is a fundamental component of viral infections, tightly linked to virus production and linked pathologies. For example, virus-induced metabolic rewiring contributes to cardiac disorders and cancers associated with infections [104,116]. An accumulating body of knowledge points to protein PTMs as critical switches in numerous metabolic pathways, and several therapeutic drugs have been developed to target enzymes that regulate PTMs. For instance, Vorinostat is a class I and II histone deacetylase (HDAC) inhibitor that is efficacious against cutaneous T cell lymphoma [301], and Onureg is used to treat acute myeloid leukemia by preventing DNA methylation through inhibition of DNA methyltransferase [302]. Mitochondrial sirtuins have also been pharmacologically targeted with small molecules in metabolic and neurodegenerative diseases [303]. Additionally, an ever-increasing diversity of modifications are being identified within the mitochondria, including numerous acylations with yet unknown functions. These findings suggest that PTMs can offer molecular signatures of mitochondrial health and cellular state. Clearly, there are still many unknown facts regarding the regulation and function of mitochondrial PTMs. However, their diversity points to a remarkably multifaceted ability for mitochondrial PTMs to contribute to rapid sensing and responses to cellular cues. Advances in mass spectrometry-based detection and quantification methods promise to help to untangle the plethora of enzymatic and non-enzymatic PTMs initiated by viral infections. Given the known viral mimicry of cellular regulatory mechanisms, these infection-induced PTM profiles are likely to uncover regulatory processes more broadly relevant in health and disease, opening avenues for therapeutic intervention.

## Figures and Tables

**Table 1:** PTMs that modulate mitochondrial functions.

Substrate	Modification	Site	Function	References
AceCS2	Acetylation	Lys642	Inhibits enzymatic activity	[54]
DRP1	Phosphorylation	Ser579, Ser600, Ser616; Ser637	Activates DRP1 binding to mitochondria; inhibits DRP1 binding	[54,55,56,57,58,59,60,61]
	S-nitrosylation	Cys644	Inhibits DRP1 oligomerization	[62]
GLS	Succinylation	Lys164	Inhibits enzymatic activity	[63]
GLUD1	Glutarylation	Lys545	Inhibits enzymatic activity	[64]
IDH2	Acetylation	Lys413	Inhibits enzymatic activity	[65]
	Succinylation	Lys360	Inhibits enzymatic activity	[66]
LCAD	Acetylation	Lys42	Inhibits enzymatic activity	[67]
MAVS	Polyubiquitination	Lys10, Lys311, Lys461	Promote MAVS aggregation	[68]
	Succinylation	Lys7	Promotes MAVS aggregation	[69]
	Phosphorylation	Ser442	Activates IRF3, innate immune signaling	[33,70]
MCD	Acetylation	Lys471	Activates enzymatic activity	[71]
MFN1	Phosphorylation	Thr562	Inhibits mitochondrial fusion	[72]
MFN2	Phosphorylation	Ser65, Thr111, Ser442	Inhibit mitochondrial fusion	[73,74]
OPA1	Acetylation	Lys834, Lys931	Inhibit mitochondrial fusion	[49]
PDHA1	Phosphorylation	Ser203, Ser264, Ser271	Inhibit enzymatic activity	[75,76,77,78]
	Lipoylation	Lys97, Lys132, Lys259	Activate enzymatic activity	[79,80]
SDH	Malonylation	Lys179	Activates enzymatic activity	[81]
VDAC1	Phosphorylation	Ser104; Ser193	Promotes cytochrome c release; inhibits cytochrome c release	[82,83,84]
	Ubiquitination	Lys274	Inhibits apoptotic signaling	[85]

## Data Availability

Not applicable.
